# Treatment Resistant Depression (TRD) and treatment response of repetitive Transcranial Magnetic Stimulation (rTMS)

**DOI:** 10.1192/j.eurpsy.2025.1329

**Published:** 2025-08-26

**Authors:** L. Holst, M. Speed, P. Kølbæk, M. Sørensen, M. B. K. Pedersen, S. P. Alves, C. Gasse

**Affiliations:** 1 Department of Affective Disorders, Aarhus University Hospital - Psychiatry; 2 Department of Clinical Medicine, Aarhus University; 3 Psychosis Research Unit, Aarhus University Hospital - Psychiatry, Aarhus; 4 Psychiatry, Randers Regional Hospital, Randers; 5 Psychiatry, Regional Psychiatry Central Jutland, Viborg, Denmark

## Abstract

**Introduction:**

Treatment-Resistant Depression (TRD) is commonly defined as an inadequate response to two or more antidepressant trials. The severity of TRD can be evaluated by means of the Maudsley Staging Method (MSM). The MSM consists of five domains including duration of the depression, symptom severity, number of treatment failures, augmentation therapy, and electroconvulsive therapy use. The Danish Medicines Council has suggested that the MSM could guide treatment decisions. However, the use of MSM in clinical practice is lagging and the evaluation of TRD in clinical practice has remained subjective and unstandardized.

**Objectives:**

To evaluate the use of MSM in clinical practice, we investigate the association between MSM scores and response to repetitive transcranial magnetic stimulation (rTMS) treatment, as measured by the 17-item Hamilton Depression Rating Scale (HAM-D17).

**Methods:**

Patients with unipolar depression referred to six weeks of treatment with rTMS were consecutively invited to participate in the study. The study was a multi-center study with inclusion sites in Randers, Viborg, and Skejby, Denmark. Demographic information and clinical data were retrieved from the patient’s Electronic Health Record (EHR). The patients were assessed on the MSM within the first two weeks of the treatment. Ratings on the HAM-D17 and self-administered questionnaires including the depressive symptoms, side effects, and quality of life were completed on a weekly basis. Additionally, three side-effect interview assessments were conducted during the six weeks rTMS treatment. A power calculation estimated the inclusion of 65 patients.

Response is to be evaluated by means of the Spearman’s rank correlation coefficient (rho) between MSM score and HAM-D17. An adequate response is defined as a 50% decrease in the Hamilton score or below the threshold for remission.

**Results:**

Data collection is ongoing. As of September 2024, a total of 59 out of 65 participants have been recruited. Preliminary results will be presented at the EPA 2025 Congress.

**Conclusions:**

A systematic assessment of TRD according to the MSM in clinical practice, holds promise for predicting treatment outcomes and guiding treatment decisions. Integrating MSM, for instance, during the recording of patient medical history, could enhance its practical utility in everyday clinical settings.

**Disclosure of Interest:**

None Declared

